# The prevalence, patterns, and correlates of gambling behaviours in men: An exploratory study from Goa, India

**DOI:** 10.1016/j.ajp.2019.03.021

**Published:** 2019-06

**Authors:** Urvita Bhatia, Bhargav Bhat, Sanju George, Abhijit Nadkarni

**Affiliations:** Sangath, H No 451 (168) Bhatkar Waddo, Porvorim, Socorro 403501, India

**Keywords:** Gambling, Epidemiology, Addictions research, India

## Abstract

**Background:**

There is a significant evidence gap on gambling in India, where gambling is viewed predominantly through the legal lens. The aim of this study is to determine the prevalence, patterns, and correlates of gambling.

**Methods:**

Cross-sectional data from the follow-up assessment in a cohort (n = 1514 men) from India. The following data were collected using a structured questionnaire: socio-demographic information, gambling, interpersonal violence, tobacco use, alcohol use disorders (AUD), common mental disorders, and suicidality. Logistic regression models were used to examine the correlates of gambling.

**Results:**

658 participants (45.4%) reported gambling in the past year, and lottery was the most frequent form of gambling (67.8%). Current gambling was correlated with rural residence (OR 1.42, CI 1.05–1.93, p = 0.02), work-related problems (OR 1.42, CI 1.03–1.96, p = 0.03), interpersonal violence (OR 3.45, CI 1.22–9.75, p = 0.02), tobacco use (OR 1.59, CI 1.16–2.19, p = 0.004), and AUD (OR 2.14, CI 1.35–3.41, p = 0.001). 724 (49.9%) participants reported gambling at least once in their lifetime. Lifetime gambling was correlated with work-related problems (OR 1.57, CI 1.14–2.17, p = 0.006), interpersonal violence (OR 4.03, CI 1.32–12.30 p = 0.02), tobacco use (OR 1.60, CI 1.16–2.20, p = 0.004), and AUD (OR 2.12, CI 1.33–3.40, p = 0.002). Age was significantly associated with playing lottery more frequently (OR 3.24, CI 1.34–7.84, p = 0.009) and tobacco use was significantly associated with playing matka more frequently (OR 1.69, CI 1.08–2.64, p = 0.02).

**Discussion:**

The high prevalence of gambling and its association with social problems and risk factors for non-communicable diseases warrants further epidemiological research.

## Highlights

• First large community study from India examining the epidemiology of gambling.
• Almost half adult men engage in gambling, with a third engaging in multiple forms.
• Gambling is associated with adverse clinical correlates, of public health importance.

## 1 Background

Gambling behaviours and their associated impact mimic those of other addictions: loss of control, tolerance to the level of activity, withdrawal symptoms, and negative consequences at an individual and broader level (Prakash et al., 2012; Rash et al., 2016). ‘Pathological gambling’, according to the International Classification of Diseases (ICD) 10, is ‘a disorder characterized by a preoccupation with gambling and the excitement that gambling with increasing risk provides’ (World Health Organization, 1992). In the latest Diagnostic and Statistical Manual of Mental Disorders (DSM-5), gambling-related problems were reclassified from ‘Impulse Control Disorder’ to ‘Substance-related and Addictive Disorders’ (American Psychiatric Association, 2013). This shift implies commonalities between gambling and other addictions, and include common or similar etiological factors, similar diagnostic criteria, progression, impact, and treatments (Rash et al., 2016).

In Western settings such as the United States, the estimated lifetime prevalence of gambling addiction is less than 2%, and is associated with gender, age, economic status, marital status (Rash et al., 2016), substance use disorders, risky behaviours, and depression (Prakash et al., 2012; Rash et al., 2016). Despite the relatively low prevalence rate, gambling addiction can cause a significant burden in societies and is associated with far-reaching impact on the individual, family, and social structures (George et al., 2014). This includes loss of relationships, and economic opportunities (Rash et al., 2016), physical health concerns (Prakash et al., 2012), and emotional, financial and social strain on family members (Mathews and Volberg, 2013).

Gambling has been an ever-present behaviour in Indian culture, with literature, including ancient scriptures, alluding to its nature and impact (Benegal, 2013; Bhide, 2007). Contemporary India, a rapidly-developing nation with a flourishing economy, technology advancements, and changing lifestyle choices, has seen a massive boom in the gambling industry. However, India’s current legislative framework in relation to gambling is ridden with challenges including implementation barriers, outdated laws, and lack of regulation and control over gambling practices (Benegal, 2013). Most forms of gambling- except for games of ‘skill’ such as card games, lotteries, and casinos (in some states only)- are illegal in India (Public Gaming Act India, 1867). The most popular illegal form of gambling in India is betting on outcomes of sports events (Benegal, 2013). The epidemiological study of gambling as an addiction is yet to garner attention and importance in India (George et al., 2017).

Broadly speaking, there has been an absence of systematic research and clinical perspectives relevant for the Indian setting (Benegal, 2013; George et al., 2014). Only three studies until date have focused on gambling in India, which have been conducted in diverse populations such as school/college students and professionals. In the first study, 79% psychiatrists reported to have seen individuals with gambling-related problems in their clinical practice; and highlighted the challenge of limited expertise in the treatment of such problems (George et al., 2014). The other two studies found the prevalence of lifetime gambling to be 19.5% and problematic gambling to be 7.4% in college students, and 27.9% and 7.1% respectively in school students (George et al., 2016; Jaisoorya et al., 2017). There have been no population level studies examining the prevalence and correlates of gambling in India.

The objective of our study was to examine the prevalence, patterns, and correlates of gambling behaviours in men in a community sample. Our hypothesis was that gambling behaviours would be associated with health problems, other addictive behaviours, and social problems.

## 2 Methods

### 2.1 Setting

The study was conducted in Goa, a small state on the West coast of India, with a population of approximately 1.4 million people. Goa ranks high on various socio-economic parameters in comparison with other states in India, with 62% of its populations being urban dwellers, a sex ratio of 973 females per 1000 males and average literacy rate of 90% (Government of India, 2011).

Some forms of gambling are legal in Goa, including state-run lotteries and privately-run off-shore and land-based casinos. The former fall under the jurisdiction of the Directorate of Small Savings and Lotteries in Goa, and its revenue is channeled to social welfare causes in the state. The latter have served to boost Goa’s tourism industry, with an expanding casino market (currently there are 15 operational casinos), seeing around 15,000 visitors per day, which is expected to increase by 30% annually. The total revenue from casinos in Goa was estimated to be $20 million dollars in 2013. The issue around casinos in Goa is complex, with some in favor of denying entry to locals, with the intention of reducing its burden in the local population (George et al., 2017; Szybala, 2016).

### 2.2 Study design

In 2006–2008, 1899 men were recruited for a cross-sectional survey using a two-stage probability sampling procedure in urban and rural communities of North Goa (Pillai et al., 2013). The population-based sample was selected based on electoral rolls, and participants were selected at random from eligible households, which were randomly selected as well. The refusal rate for randomly selected households was 1.5%. From 2012 to 2014 these men were interviewed again and a range of outcomes were measured- the data presented in this study is from the follow-up survey. The total number of males who completed the follow-up survey were 1514 (79.7%) and they form the sample for this study (Nadkarni et al., 2016).

### 2.3 Measurements

Consenting participants were administered the self-report questionnaire by trained research workers. The research workers were blind to the hypotheses to avoid non-random misclassification of outcomes. Quality control procedures included re-interviewing randomly selected participants by the research coordinator, and random visits by the research coordinator to directly observe the research workers administering the questionnaire. The measurement tools underwent rigorous translation and back-translation procedures, and field-testing in a pilot phase prior to actual data collection.

The following data were collected during the follow up survey: a) Socio-demographic information. b) Work-related problems: assessed through the following questions: 1) ‘*How often did you do no work at times when you were supposed to be working*?’ 2) ‘*How often did you find yourself not working as carefully as you should?*’ 3) ‘*How often was the quality of your work lower than it should have been*?’ 4) ‘*How often did you not concentrate enough on your work*?’ 5) ‘*How often did health problems limit the kind or amount of work you could do*?’ Work-related problems were determined if there was a positive response to one or more of these questions. c) Interpersonal violence: assessed through the following questions: ‘*In the past 12 months have you slapped, hit, kicked, punched your wife/partner or done something else that did or could have hurt her physically*?’ ‘*In the past 12 months, have you had sex with your wife/partner when he/she was unwilling or force him/her to do sexual things or to have sex*?’ d) Current tobacco use: assessed by use of tobacco (smoked and/or chewed) in the past 12 months. e) Alcohol Use Disorder (AUD): assessed by the Alcohol Use Disorders Identification Test (AUDIT), which has been validated in India and used extensively in the study setting (Nadkarni et al., 2017; Pal et al., 2004; Saunders et al., 1993). The cutoff score for the diagnosis of AUD was ≥8. f) Common Mental Disorder (CMD): assessed using the General Health Questionnaire (Goldberg, 1978), which has been widely used in the study setting (Patel et al., 2008). g) Suicidality: assessed using the Mini International Neuropsychiatric Interview (MINI 6.0) (Sheehan et al., 1998), which has been validated and used extensively in India (Salve et al., 2012). Current suicidality (past one month) was determined by at least one positive response of ‘Yes’ to any of the suicidality items. h) Gambling behaviours: Data about gambling was collected through the following questions: 1) ‘*Have you ever gambled in your lifetime?*’ 2) ‘*If yes, in how many times in the last 12 months, did you a) play lotteries or numbers, b) play ‘matka’ (*where slips are pulled from a large earthenware pot known as a matka, and random numbers are generated*), c) play card games for money, d) play dice games for money, e) go to a casino to gamble, f) play lotto for money, g) bet on shares market/stocks and/or commodities market, h) bet on sports, i) bet on animal sports such as bullfighting, j) play slot machines or other gambling machines, k) play any other game for money.*’ The responses were captured using a Likert scale, where 0= not at all in the last 12 months, 1= less than once a month, 2= once a month, 3= once a week, 4 = 2 or 3 times a week, and 5= every day or nearly every day. The participants were categorised as either current gamblers (anyone gambling at least once in the past year) or non-current gamblers, as well as lifetime gamblers or non-lifetime gamblers.

### 2.4 Ethics

Ethical approval to conduct the study was obtained from the local Institutional Review Board at the host institute (Sangath), a national ethics committee (Indian Council of Medical Research), and an international ethics committee (London School of Hygiene and Tropical Medicine). The research workers who collected the data completed the NIH Protecting Human Research Participant online course before the start of data collection. Participants diagnosed with AUD or CMD were offered free clinical assessment and treatment by a psychiatrist.

### 2.5 Analyses

Prevalence rates and patterns of gambling were calculated as proportions and means as appropriate. Socio-demographic characteristics were described as proportions and means as appropriate, for the full sample and compared between gamblers and non-gamblers using chi square test and t test, as appropriate. The association between lifetime and current gambling and the socio-demographic/clinical correlates was calculated as odds ratios (OR) using univariable logistic regression. All variables associated with lifetime and current gambling at p < 0.1 on univariable logistic regression were included in a multivariable model and variables were then excluded one by one until all remaining variables were independently associated with the outcome at p < 0.05. The same approach was followed to examine the association between more frequent forms of current gambling and socio-demographic/clinical correlates. For the commonest forms of gambling (i.e. lottery and matka), the average time spent in gambling was recategorised- “up to once a month” was recoded as “not frequent” and “more than once a month and up to thrice a week” and “more than thrice a week” was recoded as “frequent”. All associations were reported as OR and 95% confidence intervals. All the analyses were performed using Stata version 13.

## 3 Results

### 3.1 Socio-demographic characteristics (Table 1)

Mean age of the participants was 40.26 years (SD 8.94). Majority of the participants were from rural areas (61.5%), married/cohabiting (77.4%), employed (89.2%), and had completed some form of education (95.4%).

**Table 1:** Socio demographic and other correlates of gambling.

	Total n (%) n = 1451	Lifetime Gamblers n (%) n = 724 (49.9%)	Lifetime Non-gamblers n (%) n = 727 (50.1%)	Current Gamblers n (%) n = 658 (45.4%)	Current Non-gamblers n (%) n = 793 (54.7%)
Socio-demographic details					
Age					
23–39 years	711 (49%)	368 (50.8%)	343 (47.2%)	328 (49.8%)	383 (48.3%)
40 years and above	740 (51%)	356 (49.2%)	384 (52.8%)	330 (50.1%)	410 (51.7%)
Marital status					
Never married/post-marital	329 (22.7%)	176 (53.5%)	153 (46.5%)	157 (47.7%)	172 (52.3%)
Married/cohabiting	1122 (77.3%)	548 (48.8%)	574 (51.2%)	501 (44.7%)	621 (55.4%)
Area of residence					
Urban	558 (38.5%)	246 (44.1%)	312 (55.9%)	211 (37.8%)	347 (62.2%)
Rural	893 (61.5%)	478 (53.5%)	415 (46.5%)	446 (49.9%)	447 (50.1%)
Education level					
No education/ informal education	67 (4.6%)	31 (46.3%)	36 (53.7%)	29 (43.3%)	38 (56.7%)
School/college	1384 (95.4%)	693 (50.1%)	691 (49.9%)	629 (45.5%)	755 (54.6%)
Employment status					
Unemployed (including students/homemakers)	157 (10.8%)	69 (43.0%)	88 (56.1%)	62 (39.5%)	95 (60.5%)
Employed	1294 (89.2%)	655 (50.6%)	639 (49.4%)	596 (46.1%)	698 (53.9%)
Other correlates					
Work-related problems^1^ (n = 1291)					
Yes	383 (29.7%)	236 (61.6%)	147 (38.4%)	220 (57.4%)	163 (42.6%)
No	908 (70.3%)	420 (46.3%)	488 (53.7%)	378 (41.6%)	530 (58.4%)
Interpersonal violence^2^(n = 910)					
Yes	25 (2.8%)	19 (76.0%)	6 (24.0%)	18 (72.0%)	7 (28.0%)
No	885 (97.3%)	436 (49.3%)	449 (50.7%)	399 (45.1%)	486 (54.9%)
Tobacco use					
Yes	473 (32.7%)	287 (60.7%)	186 (39.3%)	271 (57.3%)	202 (42.7%)
No	974 (67.3%)	435 (44.6%)	540 (55.4%)	386 (39.6%)	589 (60.4%)
*MV 4*					
Alcohol Use Disorder					
Yes	184 (12.7%)	127 (69.0%)	57 (30.0%)	120 (65.2%)	64 (34.8%)
No	1263 (87.3%)	595 (47.1%)	668 (52.9%)	537 (42.5%)	726 (57.5%)
*MV 4*					
Common Mental Disorder					
Yes	37 (2.6%)	20 (54.1%)	17 (45.0%)	19 (51.4%)	18 (48.7%)
No	1411 (97.4%)	704 (49.9%)	707 (50.1%)	639 (45.3%)	772 (54.7%)
*MV 3*					
Suicidality					
Yes	123 (8.5%)	83 (67.5%)	40 (32.5%)	79 (64.2%)	44 (35.8%)
No	1327 (91.5%)	641 (48.3%)	686 (51.7%)	579 (43.6%)	748 (56.4%)
*MV 1*					

^1^Only in employed; ^2^Only among married/cohabiting; MV = Missing values.

### 3.2 Prevalence and types of gambling (Table 2)

724 (49.9%) participants reported engaging in gambling behaviours at least once in their lifetime. 658 (45.4%) participants reported current gambling (i.e. in the past 12 months). The types of gambling are described in terms of frequency and number of gambling activities in Table 2. The most common form of gambling was the lottery (n = 446, 67.8%). The highest frequency of gambling activity was *matka* (n = 344), with approximately 39.5% participants engaging in the activity at least once to thrice a week. 484 current gamblers (73.6%) engaged in at least one form of gambling, 225 (34.2%) engaged in 2–3 forms of gambling, and 15 (2.3%) engaged in 4 or more forms of gambling.

**Table 2:** Patterns of current gambling.

	Total number (in the past year) n = 658 n (%)	Average time spent (in the past year)		
		Up to once a month n (%)	More than once a month and up to thrice a week n (%)	More than thrice a week n (%)
Lottery	446 (67.8%)	418 (93.7%)	27 (6.1%)	1 (0.2%)
Matka	344 (52.3%)	189 (54.9%)	136 (39.5%)	19 (5.5%)
Cards	50 (7.6%)	41 (82%)	7 (14%)	2 (4%)
Dice	31 (4.7%)	31 (100%)	0	0
Sports	22 (3.3%)	16 (72.7%)	4 (18.2%)	2 (9.1%)
Casino	7 (1.1%)	7 (100%)	0	0
Lotto	5 (0.8%)	5 (100%)	0	0
Other (e.g. carrom)	4 (0.6%)	1 (25%)	3 (75%)	0

### 3.3 Socio demographic and clinical correlates of lifetime and current gambling (Table 3)

On univariable logistic regression, lifetime gambling was associated with rural residence (OR 1.46, 95% CI 1.18–1.81, p < 0.001), work-related problems (OR 1.86, 95% CI 1.46–2.38, p < 0.001), interpersonal violence (OR 3.26, 95% CI 1.28–8.28, p < 0.01), tobacco use (OR 1.91, 95% CI 1.52–2.40, p < 0.001), AUD (OR 2.50, 95% CI 1.79–3.49, p < 0.001), and suicidality (OR 2.22, 95% CI 1.49–3.29, p < 0.001). On multiple logistic regression, there was significant association between lifetime gambling and work-related problems (OR 1.57, 95% CI 1.14–2.17, p = 0.006), interpersonal violence (OR 4.03, 95% CI 1.32–12.30, p = 0.02), tobacco use (OR 1.60, 95% CI 1.16–2.20, p = 0.004), and AUD (OR 2.12, 95% CI 1.33–3.40, p = 0.002).

**Table 3:** Socio demographic and other correlates of lifetime and current gambling.

	Lifetime gambling				Current gambling			
	OR (CI) Univariable	p	OR (CI) Multivariable	p	OR (CI) Univariable	p	OR (CI) Multivariable	p
Socio-demographic details								
Age								
23-39 years	1				1			
40 years and above	0.86	0.16			0.94 (0.76-1.15)	0.55		
Marital status								
Never married/post-marital	1				1			
Married/cohabiting	0.83 (0.65-1.06)	0.14			0.88 (0.69- 1.13)	0.33		
Area of residence								
Urban	1		1		1		1	
Rural	1.46 (1.18-1.81)	0.0005	1.29 (0.96-1.74)	0.092	1.65 (1.33- 2.05)	<0.001	1.42 (1.05-1.92)	0.02
Education level								
No education/ informal education	1				1			
School/college	1.16 (0.71- 1.90)	0.54			1.09 (0.67-1.79)	0.73		
Employment status								
Unemployed (including students/homemakers)	1				1			
Employed	0.76 (0.55-1.07)	0.11			0.76 (0.54- 1.07)	0.12		
Other correlates								
Work-related problems^1^ (n = 1293)								
No	1		1		1		1	
Yes	1.87 (1.46-2.39)	<0.001	1.52 (1.10-2.10)	0.011	1.89 (1.48- 2.42)	<0.001	1.42 (1.03-1.96)	0.03
Interpersonal violence^2^(n = 1122)								
No	1		1		1		1	
Yes	3.26 (1.28- 8.28)	0.008	3.88 (1.27-11.90)	0.018	3.13 (1.29- 7.60)	0.008	3.45 (1.22-9.75)	0.02
Tobacco use								
No	1		1		1		1	
Yes	1.92 (1.53- 2.40)	<0.001	1.60 (1.16-2.21)	0.004	2.05 (1.63- 2.57)	<0.001	1.59 (1.16-2.19)	0.004
*MV 3*								
Alcohol Use Disorder								
No	1		1		1		1	
Yes	2.50 (1.79- 3.50)	<0.001	2.03 (1.26-3.27)	0.003	2.53 (1.83- 3.52)	<0.001	2.14 (1.35-3.41)	0.001
*MV 4*								
Common Mental Disorder								
No	1				1			
Yes	1.18 (0.61-2.28)	0.62			1.28 (0.66-2.45)	0.46		
*MV 3*								
Suicidality								
No	1		1		1		1	
Yes	2.22 (1.50-3.30)	<0.001	1.73 (0.89-3.36)	0.106	2.32 (1.58-3.42)	<0.001	1.67 (0.88-3.18)	0.12
*MV 1*								

^1^Only in employed; ^2^Only among married/cohabiting; MV = Missing values.

On univariable logistic regression, current gambling was associated with rural residence (1.65, 95% CI 1.33–2.05, p < 0.001), work-related problems (OR 1.89, 95% CI 1.48–2.42, p < 0.001), interpersonal violence (OR 3.13, 95% CI 1.29–7.60, p = 0.008), tobacco use (OR 2.05, 95% CI 1.63–2.57, p < 0.001), AUD (OR 2.53, 95% CI 1.83–3.52, p < 0.001), and suicidality (OR 2.32, 95% CI 1.58–3.42, p < 0.001). On multiple logistic regression, there was significant association between current gambling and rural residence (OR 1.42, 95% CI 1.05–1.93, p = 0.02), work-related problems (OR 1.42, 95% CI 1.03–1.96, p = 0.03), interpersonal violence (OR 3.45, 95% CI 1.22–9.75, p = 0.02), tobacco use (OR 1.59, 95% CI 1.16–2.19, p = 0.004), and AUD (OR 2.14, 95% CI 1.35–3.41, p = 0.001).

### 3.4 Socio demographic and clinical correlates of more frequent gambling (Table 4, Table 5)

For the more common forms of gambling, i.e. lottery and matka, we examined the associations between more frequent current gambling (defined as gambling more than once a month) and socio-demographic and clinical correlates. In comparing the two forms of gambling, 6.3% of current lottery gamblers and 45.1% of current matka gamblers played more than once a month. Age and work-related problems were associated with more frequent playing of lottery on univariable analysis. However, only age retained significant association with more frequent playing of lottery, with those 40 years and above more likely to play lottery more frequently (OR 3.24, 95% CI 1.34–7.84, p = 0.009). Tobacco use, AUD and suicidality and frequent playing of matka were found to be associated on univariable analysis. On multivariable analysis, only tobacco use was significantly associated with playing matka more frequently (OR 1.69, 95% CI 1.08–2.64, p = 0.02) ().

**Table 4:** Socio demographic and other correlates of two common types of gambling.

	Total n (%) n = 446	Playing lottery less than once a month n (%) n = 418 (93.7%)	Playing lottery more than once a month n (%) n = 28 (6.3%)	Total n (%) n = 344	Playing matka less than once a month n (%) n = 189 (54.9%)	Playing matka more than once a month n (%) n = 155 (45.1%)
Socio-demographic details						
Age						
23-39 years	219 (49.1%)	212 (96.8%)	7 (3.2%)	170 (49.4%)	88 (51.8%)	82 (48.2%)
40 years and above	227 (50.9%)	206 (90.7%)	21 (9.3%)	174 (50.6%)	101 (58%)	73 (42%)
Marital status						
Never married/post-marital	109 (24.4%)	105 (96.3%)	4 (3.7%)	80 (23.3%)	46 (57.5%)	34 (42.5%)
Married/cohabiting	337 (75.6%)	313 (92.9%)	24 (7.1%)	264 (76.7%)	143 (54.2%)	121 (45.8%)
Area of residence						
Urban	153 (34.3%)	144 (94.1%)	9 (5.9%)	102 (29.7%)	60 (58.8%)	42 (41.2%)
Rural	293 (65.7%)	274 (93.5%)	19 (6.5%)	242 (70.3%)	129 (53.3%)	113 (46.7%)
Education level						
No education/ informal education	13 (2.9%)	12 (92.3%)	1 (7.7%)	19 (5.5%)	7 (36.8%)	12 (63.2%)
School/college	433 (97.1%)	406 (93.8%)	27 (6.2%)	325 (94.5%)	182 (56%)	143 (44%)
Employment status						
Unemployed (including students/homemakers)	32 (7.2%)	32 (100%)	0	43 (12.5%)	23 (53.5%)	20 (46.5%)
Employed	414 (92.8%)	386 (93.2%)	28 (6.8%)	301 (87.5%)	166 (55.1%)	135 (44.9%)
Other correlates						
Work-related problems^1^ (n = 1291)						
Yes	149 (36%)	143 (96%)	6 (4%)	124 (41.2%)	67 (54%)	57 (46%)
No	265 (64%)	243 (91.7%)	22 (8.3%)	177 (58.8%)	98 (55.4%)	79 (44.6%)
Interpersonal violence^2^(n = 910)						
Yes	13 (4.7%)	12 (92.3%)	1 (7.7%)	9 (4%)	6 (66.7%)	3 (33.3%)
No	263 (95.3%)	247 (93.9%)	16 (6.1%)	216 (96%)	114 (52.8%)	102 (47.2%)
Tobacco use						
Yes	153 (34.4%)	140 (91.5%)	13 (8.5%)	193 (56.1%)	93 (48.2%)	100 (51.8%)
No	292 (65.6%)	277 (94.9%)	15 (5.1%)	151 (43.9%)	96 (63.6%)	55 (36.4%)
Alcohol Use Disorder						
Yes	69 (15.5%)	62 (89.9%)	7 (10.1%)	97 (28.2%)	42 (43.3%)	55 (56.7%)
No	376 (84.5%)	355 (94.4%)	21 (5.6%)	247 (71.8%)	147 (59.5%)	100 (40.5%)
Common Mental Disorder						
Yes	11 (2.5%)	11 (100%)	0	16 (4.6%)	9 (56.3%)	7 (43.7%)
No	435 (97.5%)	407 (93.6%)	28 (6.4%)	328 (95.4%)	180 (54.9%)	148 (45.1%)
Suicidality						
Yes	48 (10.8%)	45 (93.7%)	3 (6.3%)	62 (18%)	27 (43.5%)	35 (56.5%)
No	398 (89.2%)	373 (93.7%)	25 (6.3%)	282 (82%)	162 (57.4%)	120 (42.6%)

^1^Only in employed; ^2^Only among married/cohabiting.

**Table 5:** Socio demographic and clinical correlates of more frequent gambling.

	Frequent lottery				Frequent matka			
	OR (CI) Univariable	p	OR (CI) Multivariable	p	OR (CI) Univariable	p	OR (CI) Multivariable	p
Socio-demographic details								
Age								
23-39 years	1		1		1			
40 years and above	3.09 (1.27-7.48)	0.008	3.24 (1.34- 7.84)	0.009	0.77 (0.51- 1.19)	0.24		
Marital status								
Never married/post-marital	1				1			
Married/cohabiting	2.01 (0.68- 5.95)	0.197			1.14 (0.69- 1.90)	0.600		
Area of residence								
Urban	1				1			
Rural	1.11 (0.49- 2.52)	0.803			1.25 (0.78- 2.00)	0.348		
Education level								
No education/ informal education	1				1			
School/college	0.80 (0.10- 6.38)	0.831			0.46 (0.18- 1.20)	0.103		
Employment status								
Unemployed (including students/homemakers)	1				1			
Employed	0	0.129			1.07 (0.56- 2.03)	0.838		
Other correlates								
Work-related problems^1^ (n = 1293)								
No	1		1		1			
Yes	0.46 (0.18- 1.17)	0.096	0.44 (0.17-1.13)	0.08	1.06 (0.67- 1.67)	0.819		
Interpersonal violence^2^(n = 1122)								
No	1				1			
Yes	1.29 (0.16- 10.57)	0.814			0.56 (0.14- 2.30)	0.414		
Tobacco use								
No	1				1		1	
Yes	0.81 (0.79- 3.71)	0.16			1.88 (1.21- 2.92)	0.004	1.69 (1.08- 2.64)	0.02
Alcohol Use Disorder								
No	1				1		1	
Yes	1.91 (0.78- 4.69)	0.152			1.93 (1.19- 3.11)	0.006	1.61 (0.98- 2.65)	0.06
Common Mental Disorder								
No	1				1			
Yes	0	0.385			0.95 (0.34- 2.60)	0.914		
Suicidality								
No	1				1		1	
Yes	0.99 (0.28- 3.43)	0.993			1.75 (1.00- 3.06)	0.046	1.47 (0.83- 2.62)	0.19

1Only in employed; ^2^Only among married/cohabiting.

## 4 Discussion

We examined the prevalence and correlates of gambling in a large community-based sample of adult men in Goa. We found a high prevalence of current gamblers (past 12 months) in the sample, with close to half (45.4%) reporting engaging in some kind of gambling activity, and one-third of those who gamble engaging in multiple forms of gambling. We also found that lifetime and current gambling are associated with work-related problems, interpersonal violence, tobacco use and alcohol use disorders; and additionally, current gambling is associated with rural residence. These are important findings, especially given the dearth of research evidence exploring gambling behaviours in India.

Research on the prevalence of current gambling worldwide has highlighted wide variations across and within settings, from 76.9% to 82.2% in the US, to 41.8% to 81.1% in Asia, 64% to 86% in Oceania, and 25.5% to 80.6% in Europe (Calado and Griffiths, 2016). The prevalence of current gambling in our sample is higher than the prevalence rate found in another study from India. In a cross-sectional survey with college students in Kerala (George et al., 2016), 19.5% of participants reported having ever gambled, with the prevalence of problem gambling being 7.4% amongst those who gamble. In the same study, majority of participants played lottery, which was found in our sample as well. With increasingly easy access to gambling outlets in India, there is an anticipated increase in the number of people engaging in gambling and the proportion who will then go on to become problem gamblers (George et al., 2017). Given the lack of exploration in this area, future studies could potentially examine trends in gambling behaviours, and protective and risk factors associated with gambling in India (Benegal, 2013).

Our findings on the correlates of gambling are consistent with global literature on comorbidities associated with gambling, particularly problem gambling, including perpetrating violence, and clinical conditions such as substance use disorders and related problems (Dowling et al., 2014; George et al., 2016; Jaisoorya et al., 2017; Mann et al., 2017; Rash et al., 2016). From the two most common forms of gambling, it is interesting to note that matka, despite being an illegal form of gambling was played more frequently, with majority of current matka gamblers playing more than once a month. Clinical interventions targeting gambling need to take into consideration factors that increase the odds of becoming a more frequent gambler, including age (i.e. being older and using other substances (i.e. such as tobacco).

Overall, gambling behaviours and addictions are issues that are largely overlooked in clinical practice and research priorities in India. Given the commonalities in etiology and characteristics between pathological gambling and other addiction-related problems, the former should not be viewed independently in research and clinical inquiry (Rash et al., 2016). Also, future research is needed to examine trends in the prevalence of gambling disorders, given its relation with social and legal sanctions. In India, the public response to gambling has been largely shaped by moral and legal perspectives; and these frameworks undermine other perspectives, particularly a clinical one. Such a scenario necessitates the development and advancement of epidemiological research exploring the burden of gambling and pathways to help, with the end goal being to guide public and legal responses, in addition to clinical interventions to prevent and treat gambling related problems (Benegal, 2013; George et al., 2017).

The major strengths of our study are its sample size, use of structured interviewing tools, and the blinding of research workers to the proposed hypothesis. The limitations of our study include the lack of generalisability of our findings to both genders, the potential under-reporting of gambling behaviours because of social desirability, and the inability to examine direction of causality between gambling behaviours and the various correlates because of nature of the study design .

In conclusion, less than half adult men engage in gambling behaviours, a finding that has public health implications considering that help-seeking for addictions and accessible formal sources of help are minimal. Furthermore, there is a need to examine explanatory models and pathways to help to reduce unmet needs of the target population; a greater understanding of the short and long-term burden of gambling on a range of outcomes; and mechanisms that can be targeted in prevention and treatment programs. This accumulated evidence needs to be used to influence the decision-making of clinical practitioners as well as policy makers.

## Financial disclosure

This work was supported by the Wellcome Trust Research Training Fellowship to Abhijit Nadkarni [grant number WT093897MA]. Urvita Bhatia was supported by a Wellcome Trust DBT India Alliance Research Training Fellowship [grant number IA/RTF/15/1/1018]. The funding agencies had no role in the analysis and interpretation of data; in the writing of the manuscript; or in the decision to submit the paper for publication.

## Conflicts of interest

None.
